# Association of hereditary thrombophilia with intrauterine growth restriction

**Published:** 2013-04

**Authors:** Fatemeh Mirzaei, Zohreh Farzad-Mahajeri

**Affiliations:** 1 *Department of Obstetrics and Gynecology, Physiology Research Center, Kerman University of Medical Sciences, Kerman, Iran. *; 2 *Department of Obstetrics and Gynecology, Afzalipour Hospital, Kerman University of Medical Sciences, Kerman, Iran.*

**Keywords:** *Intra uterine growth retardation*, *Hereditary*, *Thrombophilia*, *Pregnancy*

## Abstract

**Background:** Intrauterine growth retardation (IUGR) contributes significantly to fetal morbidity and mortality, but its etiology is unknown in most cases.

**Objective:** The aim of this study was to examine the association between inherited thrombophilia and IUGR.

**Materials and Methods:** A case-control study was performed in a tertiary referral center (Afzalipour Hospital) over 2-years period (2010-2011). Cases (n=25) were women who had pregnancies complicated by IUDR and control subjects (n=25) were women who had normal growth fetuses. All women were tested for inherited thrombophilia at least 4 weeks after delivery. Main outcome measure was prevalence of maternal thrombophlia. Genotyping for factor V Leiden, prothrombin gene (nucleotide G20210A), and MTHFR (C677T) mutation was performed by PCR technique. Protein C, S and antithrombin III activity were determined with a clotting assay (STA-Staclot, France).

**Results:** The prevalence of hereditary thrombophilia was 68% (n=17) in IUGR group, and 32% (n=8) in control group (OR: 1.5, p=0.011, 95% CI: 1.3-14.8). The frequency of MTHFR (C677T) gene mutation (p=0.037; OR: 3.69) and protein S deficiency (p=0.034; OR: 5.41) was significantly increased in the group with IUGR compared with the control group. There was no significant difference between the two groups in prothrombin G20210A mutation (p=0.490) and protein C deficiency (p=0.609). A significant difference in the frequency of multiple thrombophilias was detected between the two groups (p=0.009).

**Conclusion:** This study revealed that protein S deficiency and MTHFR gene mutation are more prevalent in pregnancies with IUGR.

## Introduction

Successful pregnancy outcome is dependent on the development and maintenance of adequate placental circulation. Abnormalities of placental vasculature may result in a number of gestational pathologies, such as intrauterine growth restriction (IUGR) (1, 2). IUGR is a frequent cause of stillbirth, perinatal morbidity, and long term sequels, but its etiology is unknown in most cases (3, 4). It has been suggested to be associated with abnormal placental vascular and disturbance of homeostasis leading to inadequate maternal-fetal circulation (5).

There is a growing view that inherited thrombophilia may pre-dispose to adverse pregnancy outcome (6, 7). The probable mechanism may be associated with pathological placental vascular leading to inadequate fetomaternal circulation (8). The relationship between IUGR and hereditary thrombophilia is controversial (5).

Inherited thrombophilias are a heterogeneous group of coagulation disorders that predispose individuals to thrombosis, and include mutations of the factor V Leiden, prothrombin G20210A gene mutation, methylenetetrahydrofolate reductase (MTHFR) gene mutation, antithrombin III deficiency and protein C, S deficiencies (8).

IUGR is a frequent cause of perinatal morbidity and mortality, so if we demonstrate the relationship between hereditary thrombophilia and IUGR, we can improve the prognosis of pregnancy by using therapeutic strategies in early stages. Some studies showed association between inherited thrombophilia and complications, such as interauterine fetal death, preeclampsia and placental abruption but association between IUGR and thrombophlia is controversial (6, 7). The aim of this study was to examine the association between inherited thrombophilias and IUGR in, Kerman, Iran.

## Materials and methods

We conducted a case-control study in a tertiary referral center (Afzalipour Hospital) over 2-years period (2010-2011). This study was approved by the Ethical Committee of Kerman Medical University and informed consent was obtained from each women. In this study, we studied 25 women with singleton pregnancies complicated by IUGR with unknown placental resistances and 25 women who gave birth to normal weight newborns during the same time period. Case and control groups matched for age and parity. Gestational age was calculated from the first day of the last menstrual period and confirmed by either a first- or second-trimester ultrasound scan. When the ultrasound determined gestational age differed from that calculated from the last menstrual period more than 7 days in the first trimester or by more than 10 days in the second trimester, the ultrasound-determined gestational age was used. 

IUGR defined as fetal weight less than the 10^th^ percentile for gestational age according to the criteria of Hadlock *et al* and diagnosed by serial ultrasonograghy (9). All women with IUGR had Doppler studies as part of their routine evaluation. Placental vascular resistance was evaluated with recorded flow velocity waveforms from the umbilical arteries. A raised umbilical artery pulsatility index (PI) indicates increased placental resistance. Exclusion criteria in two groups were a history of medical diseases such as chronic hypertension, diabetes, asthma, cardiac disease, chronic renal disease and collagen vascular disease, structural fetal anomalies, hydrops fetalis, and drug abuse user. We excluded women with a history of prior thrombosis, women who screened positive for thrombophilia and treated with heparin.

Data were collected via questionnaires, history taking, physical examination and laboratory tests. Two blood samples were obtained from each of the participants of the two groups at 4 weeks postpartum. One sample was assessed for mutations in factor V Leiden (nucleotide G1691A), prothrombin gene (nucleotide G20210A) and MTHFR (C677T). 

DNA was extracted from plasma samples according to Sina Gene kit protocols. DNA concentration and quality was evaluated on agarose gel 1.5%, using gel documentation and spectrophotometer respectively. Consequently the gene of each factor was copied in thermo cycler using related primer couples and by PCR and Tag DNA polymerase. Ultimately mutation of each gene was detected by PCR-RFLP technique.

The other sample was evaluated in Blood Transfusion Organization laboratory to measure protein C, protein S and antithrombin III. For plasma testing, the frozen sample was first melt and then diluted by Owren-Koller diluting solution with proportion of 1/10. Finally STACLOT kit protocol was used for measurement tests. Normal range was 70-130% for protein C, 60-120% for protein S, and 80-120% for antithrombin III. Measurments less than normal range was defined as positive and those between normal ranges as negative. 

**Statistical analysis**

Data were analyzed by statistical software SPSS 17 using T-test, chi-square and logistic regression. For statistical analysis, the statistical software SPSS version 13.0 Windows (SPSS Inc., Chicago, IL) was used. All p-values were 2 tailed and statistical significant was defined by p≤0.05.

## Results

The two groups were similar in age (p=0.062) and gravity (p=0.127), but birth weight and gestational age of IUGR cases (1200±431, 32.5±3.5) were significantly less than controls (3250±163, 39.1±0.5) (Table I). All women in control group had normal vaginal delivery, but 72% of women in IUGR group underwent cesarean section. The prevalence of hereditary thrombophilia was 68% (n=17) in IUGR group, and 32% (n=8) in control group (OR:1.5, p=0.011, 95% CI:1.3-14.8). 24% of women in IUGR group (n=6) but none of controls showed multiple thrombophilia (OR:2.1, 95% CI:0-0.2, p=0.009). 

There were no significant associations between prothrombin gene G2021A and Factor V Leiden mutation in two groups. MTHFR gene mutation (heterozygote and homozygote pattern) in IUGR group was significantly more than control group. One case in IUGR group and three in control group demonstrated protein C deficiency. Protein S deficiency in IUGR group was significantly higher than controls. None of the participants in the two groups showed antithrombin III deficiency (Table II).

**Table I:** Demographic and obstetrics characteristics of the two groups

	**IUGR**	**Control**	p-value*
Age (mean±SD)	23.5 ± 5.5	32.5 ± 3.5	0.062
Gravity (mean±SD)	1.84 ± 0.68	1.44 ± 1.08	0.127
Birth weight (gr)	1200 ± 431	3250 ± 163	0.000
Gestational age (wk)	32.5 ± 3.5	39.1 ± 0.5	0.000

*: p<0.05 is statistically significant (T-test)

**Table II:** Prevalence of thrombophilia in the two groups

**Type of thrombophilia**	**IUGR group Number (%)**	**Control group Number (%)**	*p-value	**Odds Ratio (95%CI)
Prothrombin gene mutation	2 (8%)	0	0.490	1.5
Factor V gene mutation	0	0	-	-
MTHFR gene mutation (+/+&+/-)	12 (48%)	5 (20%)	0.037	69
Protein C deficiency	3 (12%)	1 (4%)	0.609	3.27
Protein S deficiency	8 (32%)	2 (8%)	0.034	5.41
Antithrombin deficiency	0	0	-	-
Multiple thrombophilia	6 (24%)	0	0.009	2.1

* p<0.05 is statistically significant (Chi-square test), ** logistic regression

## Discussion

The results indicate that nearly 68% of pregnant women with IUGR carried hereditary thrombophilia. IUGR is a common cause of perinatal morbidity and mortality (10). There are various factors affecting IUGR. Recent studies have suggested role of maternal thrombotic disorders in complications such as preeclampsia, placental abruption, intra uterine fetal death and IUGR (11-14). Placental thrombosis and vascular occlusion are characteristics of complicated pregnancy (15). Correlation of hereditary thrombophilia and IUGR has been suggested in many studies, but there is no accepted guideline for screening pregnant women (16).

Our study showed a high prevalence (68%) of thrombophilia in IUGR pregnancies compared with 32% in the normal group. This finding is consistent with Jamal *et al* and Kupforminc *et al* which showed a significant association between IUGR and thrombophilia (15, 16). The difference in the prevalence of MTHFR (C677T) gene mutation and protein S deficiency between two groups in our study was statistically significant. In our study MTHFR (C677T) gene mutation was the most common thrombophilia factor in two groups, following by protein S deficiency. Jamal *et al* and Kupforminc *et al* have reported a statistically significant association between MTHFR (C677T) gene mutation and protein S deficiency and IUGR (16, 17).

Protein C deficiency was detected in three women in IUGR group and one of the controls, but this difference did not reach significant level (p=0.609). In some studies protein C deficiency has been suggested to be the cause of vascular thrombosis and IUGR (18). The association between protein C deficiency and IUGR was shown in several studies but inconsistency with Jamal *et al* and Kupforminc *et al* who did not found association between protein c deficiency and IUGR (5,16-17). 

Some studies have reported that factor V Leiden mutation in European is 8.8%. While outside Europe the mutation is very rare (17). In this study factor V Leiden was not found in two groups, while Jamal *et al* demonstrated significant relation, and Kupforminc *et al* suggested Leiden as the most common type of thrombophilia in IUGR group (16, 17). This difference needs to be assessed by larger samples in different areas of Iran. 

The association between IUGR and Prothrombin gene G20210A mutation and antithrombin III deficiency are controversial (18). In the present study Prothrombin gene G20210A mutation and antithrombin III deficiency was not detected in any of the two groups. Data from a large sample are needed for conclusive results. Multiple thrombophilia had been evaluated in few studies. In our study, 6 cases with IUGR and no women in the normal group had multiple thrombophilia (OR:2.1, 95% CI:0-0.2, p=0.009). In another study in Iran, Jamal *et al* reported 8 women in IUGR group and one of the controls to have more than two multiple thrombophilia (OR:32.5, 95% CI:3.8-280.5, p=0.002) (16). 

## Conclusion

The results from the present study support high frequency of protein S and MTHFR gene mutation in pregnant women with IUGR. One major limitation which depleted the power of this study was small sample. Further investigations through larger samples and review articles are recommended, in order to design a guideline according which first and second line laboratory tests can be assigned.
